# Supplementary material for: Elevational Changes in Bacterial Microbiota Structure and Diversity in an Arthropod-Disease Vector
Source: Microb Ecol. 2021 Oct 2;84(3):868–78. doi: 10.1007/s00248-021-01879-5 (PMC9622521; doi:10.1007/s00248-021-01879-5)
Supplement: Supplementary file 1 — Supplementary file1 (PDF 601 kb) [file 248_2021_1879_MOESM1_ESM.pdf]

Supplementary Material for "Elevational changes in bacterial microbiota structure and diversity in an arthropod-disease vector"

Tuomas Aivelo, Mélissa Lemoine & Barbara Tschirren

S1: Supplementary data collection

Sampling sites and collection

Our sampling sites were situated in Eastern Switzerland in the Swiss Alps near the confluence of the Anterior and Posterior Rhine (Figure 1). We sampled on the slopes of Crap Masegn (Sagogn, Flims, Ruschein), Fulhorn (Rodels, Tomils, Feldis) and Gürgaletsch (Passug, Castiel, Praden). In the Swiss Alps the treeline varies between 1 650 – 2 450 m and our sampling sites have mean July temperature variation between 11 and 18 degrees Celsius.

Elevational gradients in Swiss Alps have been a classical study system specifically in botanical studies [1], but microbe diversity has been less studied. In the Swiss Alps, above-ground diversity appears to decrease in groups such as plants [2], butterflies [3], orthopterans [4] and below-ground diversity to increase in groups such as fungi [5] and nematodes [4] with increasing elevation.

The most important hosts for ticks are homeothermic birds and mammals. The most common rodent species throughout the sampling area and likely most common host for earlier tick life stages is the bank vole. In a previous study, we did not observe an elevational trend on small rodent diversity [6]. Bird diversity, on the other hand, has been found to decrease with elevation in the Swiss Alps [7]. Larger mammal diversity and abundance probably varies in our sampling area more due to anthropogenic influences than due to altitudinal effects.

During each sampling session, four people dragged slowly a white blanket (1 m x 1 m each) over the ground vegetation during 15 min each. Each person regularly checked for ticks on the blanket and transferred them to microtubes containing 95% ethanol. Coordinates and elevation were recorded for each site. Tick species and life stage were verified using a stereomicroscope.

Tick microbiota sequencing

DNA isolation and amplifications were performed in a laminar flow cabinet to avoid contaminations. Each tick was washed thrice with sterile water to remove surface bacteria before sterilizing it with 3% hydrogen peroxide. To remove batch effects, we randomized samples and negative controls across two plates during library preparation.

16S sequencing libraries were prepared following the Earth Microbiome 16S Illumina Amplicon protocol, using the primers

515FB: CTTTCCCTACACGACGCTCTTCCGATCTNNNNNNNNGTGYCAGCMGCCGCGGTAA
806RB: GGAGTTCAGACGTGTGCTCTTCCGATCTNNNNNNNNGGACTACNVGGGTWTCTAAT

which also contained the Illumina Miseq primer binding site [8–10]. Samples and negative controls were randomized across two plates. Each reaction contained 1 µl of template DNA with 0.5 µM forward and reverse primers, 0.3 mM dNTP, 0.4 µl KAPA HiFi HotStart polymerase (KAPA Biosystems, Basel, Switzerland) in a final volume of 20 µl. The PCR protocol started with an initial denaturation at 95 C° for 3 minutes, followed by 35 cycles of 20 seconds at 98 C°, 60 seconds at 50 C° and 30 seconds at 72 C° with final elongation step of 5 minutes at 72 C°. We ran PCR products on 2% agarose gels, cut out the bands corresponding to the amplicon size (350 bp) and eluted them in 10 µl milli-Q water. We then purified the extracted amplicons using MinElute Gel Extraction kit (Qiagen, Hilden, Germany) following the manufacturer's protocol. In second PCR step Illumina Miseq adaptors and dual indexing Miseq barcodes were incorporated into the previously amplified V4 regions. The second PCR protocol started with an initial denaturation at 95 C° for 3 minutes, followed by 15 cycles of 20 seconds at 98 C°, 60 seconds at 54 C° and 30 seconds at 72 C° with final elongation step of 5 minutes at 72 C°. Products were visualized and purified as described above. We then measured the DNA concentration of the purified product with Invitrogen Qubit 4 Fluorometer ssDNA assay kit (Thermo Fisher Scientific, Walthan, MA, USA) and created library pools by mixing equimolar amounts of purified products to reach a 4 nM library concentration. The libraries were further processed using standard normalization protocols with PhiX controls and sequenced on a Illumina MiSeq at the Functional Genomic Center Zurich using reagent kit 600cycle v3 (Illumina, San Diego, CA, USA) with a target length of 250 bp per amplicon following the manufacturer's protocol.

S2: Supplementary methods and results of diversity analysis

Alpha diversity

Table S1a: Model selection for modelling alpha diversity of tick bacterial microbiota (inverse Simpson index).

Model	AIC	cond. R^2
invsimpson ~ elevation*tick_stage*month+elevation^2, random= ~1|location	584.0	0.20
invsimpson ~ elevation*tick_stage*month, random= ~1|location	561.4	0.20
invsimpson ~ elevation+month+tick_stage+elevation:month+tick_stage:month+ elevation:tick_stage, random= ~1|location	547.0	0.15
invsimpson ~ elevation+month+tick_stage+elevation:tick_stage+tick_stage:month, random= ~1|location	527.4	0.15
invsimpson ~ elevation+month+tick_stage++tick_stage:month, random= ~1|location	530.9	0.15
invsimpson ~ elevation+tick_stage+tick_stage:month , random= ~1|location	516.3	0.15
invsimpson ~ elevation+tick_stage, random= ~1|location	522.9	0.13
**invsimpson ~ tick_stage, random= ~1|location**	**511.9**	**0.12**
invsimpson ~ 1, random= ~1|location	515.9	0.05

Table S1b: Model selection for modelling alpha diversity of tick bacterial microbiota (Faith's index).

Model	AIC	cond. R^2
phylodiv ~ elevation*tick_stage*month+elevation^2, random= ~1|location	459.8	0.21
phylodiv ~ elevation*tick_stage*month, random= ~1|location	436.2	0.21
phylodiv ~ elevation+month+tick_stage+elevation:month+tick_stage:month+ elevation:tick_stage, random= ~1|location	417.0	0.17
phylodiv ~ elevation+month+tick_stage+elevation:tick_stage+tick_stage:month, random= ~1|location	395.2	0.14
phylodiv ~ elevation+month+tick_stage+tick_stage:month, random= ~1|location	382.7	0.13
phylodiv ~ elevation+tick_stage+ month , random= ~1|location	382.6	0.13
**phylodiv ~ elevation+month, random= ~1|location**	**381.2**	**0.12**
phylodiv ~ elevation, random= ~1|location	391.2	0.08
phylodiv ~~ 1, random= ~1|location	393.6	0.04

Table S1c: Model selection for modelling alpha diversity of tick bacterial microbiota (NRI).

Model	AIC	cond. R^2
NRI ~ elevation*tick_stage*month+elevation^2, random= ~1|location	362.8	0.19
NRI ~ elevation+month+tick_stage+elevation:month+tick_stage:month+ elevation:tick_stage+ elevation^2, random= ~1|location	343.3	0.12
NRI ~ elevation+month+tick_stage+elevation:month+tick_stage:month+ elevation:tick_stage		
NRI ~ elevation+month+tick_stage+elevation:month+ elevation:tick_stage, random= ~1|location	317.2	0.13
NRI ~ elevation+month+tick_stage+ elevation:tick_stage, random= ~1|location	299.1	0.10
NRI ~ elevation+tick_stage+ elevation:tick_stage, random= ~1|location	295.5	0.19
NRI ~ tick_stage+elevation, random= ~1|location	272.6	0.06
**NRI ~ tick_stage, random= ~1|location**	**257.0**	**0.06**
NRI ~ 1, random= ~1|location	264.9	0.00

Table S1d: Model selection for modelling alpha diversity of tick bacterial microbiota (NTI).

Model	AIC	cond. R^2
NTI ~ elevation*tick_stage*month+ elevation^2, random= ~1|location	352.4	0.14
NTI ~ elevation+month+tick_stage+elevation:month+tick_stage:month+ elevation:tick_stage+elevation^2, random= ~1|location	327.2	0.14
NTI ~ elevation+month+tick_stage++tick_stage:month+ elevation:tick_stage+ elevation^2, random= ~1|location	312.4	0.14
NTI ~ elevation+month+tick_stage+elevation:tick_stage+ elevation^2, random= ~1|location	306.5	0.14
NTI ~ elevation+month+tick_stage+ elevation^2, random= ~1|location	281.1	0.12
NTI ~ elevation+tick_stage+month , random= ~1|location	254.0	0.11
NTI ~ month+tick_stage, random= ~1|location	238.7	0.11
**NTI ~ tick_stage, random= ~1|location**	**236.3**	**0.10**
NTI ~ 1, random= ~1|location	249.4	0.00

Beta diversity

Table S2a: Model selection for modelling Bray-Curtis beta diversity of tick bacterial microbiota using permutational ANOVA. The final model is highlighted in bold.

Model	Significant variables	R^2
betad~tick_stage*elevation*location*month	tick_stage, location:month	0.46
betad~tick_stage+elevation*location*month+tick_stage:elevation:location:month+tick_stage:elevation:month+tick_stage:location:month+tick_stage:elevation+tick_stage:month+tick_stage:location	tick_stage, location:month	0.44
betad~tick_stage+elevation*location*month+ tick_stage:elevation:month+tick_stage:location:month+tick_stage:elevation+tick_stage:month+tick_stage:location	tick_stage, location:month	0.42
betad~elevation+tick_stage+location*month+tick_stage:elevation:month+tick_stage:location:month+tick_stage:elevation+tick_stage:month+tick_stage:location+elevation:location:month	elevation, location, tick_stage, elevation:location	0.39
betad~elevation+tick_stage+location*month+tick_stage:elevation:month+tick_stage:elevation+tick_stage:month+tick_stage:location+elevation:location:month	tick_stage, location:month	0.39
betad~elevation+tick_stage+location+location:month+tick_stage:elevation:month+tick_stage:elevation+tick_stage:month+tick_stage:location+elevation:location:month	elevation, location, tick_stage, location:month	0.37
betad~elevation+tick_stage+location+location:month+tick_stage:elevation:month+tick_stage:elevation+tick_stage:month+tick_stage:location	elevation, location, tick_stage, location:month, elevation:tick_stage	0.35
betad~elevation+tick_stage+location+location:month+tick_stage:elevation+tick_stage:month+tick_stage:location	location, tick_stage, location:month, elevation:tick_stage	0.31
betad~elevation+tick_stage+location+location:month+tick_stage:elevation+tick_stage:month	tick_stage, location:month	0.25
betad~ elevation+location+tick_stage+location:month+tick_stage:month	location, tick_stage, location:month	0.21
betad~elevation+tick_stage+location+location:month	tick_stage	0.19
betad~elevation+tick_stage+location	tick_stage	0.13
betad~ tick_stage+location	tick_stage	0.11
**betad~ tick_stage**	**tick_stage**	**0.09**

Table S2b: Model selection for modelling Jaccard index beta diversity of tick bacterial microbiota using permutational ANOVA. The final model is highlighted in bold.

Model	Significant variables	R^2
betad~tick_stage*elevation*location*month	tick_stage, elevation, tick_stage:location	0.41
betad~tick_stage+elevation*location*month+tick_stage:elevation:location:month+tick_stage:elevation:month+tick_stage:location:month+tick_stage:elevation+tick_stage:month+tick_stage:location	tick_stage, elevation, tick_stage:location	0.37
betad~tick_stage+elevation*location*month+ tick_stage:elevation:month+tick_stage:location:month+tick_stage:elevation+tick_stage:month+tick_stage:location	tick_stage, elevation, tick_stage:elevation:month	0.33
betad~tick_stage+ location+elevation+location:month+tick_stage:elevation:month+tick_stage:location:month+tick_stage:elevation+tick_stage:month+tick_stage:location+elevation:location:month+elevation:location	tick_stage, elevation, tick_stage:location	0.33
betad~tick_stage+ location+elevation+location:month+tick_stage:elevation:month+tick_stage:elevation+tick_stage:month+tick_stage:location+elevation:location:month +elevation:location	tick_stage, elevation tick_stage:location, tick_stage:elevation:month	0.31
betad~tick_stage+ location+elevation+location:month+tick_stage:elevation:month+tick_stage:elevation+tick_stage:month+tick_stage:location+elevation:location	tick_stage, elevation, tick_stage:location	0.28
betad~tick_stage+ location+elevation+tick_stage:elevation:month+tick_stage:elevation+tick_stage:month+tick_stage:location+elevation:location	tick_stage, elevation	0.25
betad~tick_stage+ location+elevation+tick_stage:elevation+tick_stage:month+tick_stage:location+elevation:location	tick_stage, elevation	0.21
betad~tick_stage+location+elevation+tick_stage:elevation+ tick_stage:location+elevation:location	tick_stage, elevation	0.17
betad~tick_stage+location+ elevation+ tick_stage:elevation	tick_stage, elevation	0.11
betad~tick_stage+elevation+ elevation:tick_stage	tick_stage, elevation	0.09
**betad~tick_stage+elevation**	**tick_stage, elevation**	**0.07**

Table S2c: Model selection for modelling weighted UniFrac index beta diversity of tick bacterial microbiota using permutational ANOVA. The final model is highlighted in bold.

Model	Significant variables	R^2
betad~tick_stage*elevation*location*month	tick_stage, location:month	0.44
betad~tick_stage+elevation*location*month+tick_stage:elevation:location:month+tick_stage:elevation:month+tick_stage:location:month+tick_stage:elevation+tick_stage:month+tick_stage:location	tick_stage, location:month	0.40
betad~tick_stage+elevation*location*month+ tick_stage:elevation:month+tick_stage:location:month+tick_stage:elevation+tick_stage:month+tick_stage:location	tick_stage, location:month	0.37
betad~tick_stage+ location+elevation+location:month+tick_stage:elevation:month+tick_stage:location:month+tick_stage:elevation+tick_stage:month+tick_stage:location+elevation:location:month+elevation:location	tick_stage, location:month	0.35
betad~tick_stage+ location+elevation+location:month+tick_stage:elevation:month+tick_stage:elevatio	tick_stage, location, location:month	0.33

n+tick_stage:month+tick_stage:location+elevation:location:month +elevation:location		
betad~tick_stage+ location+elevation+location:month+tick_stage:elevation:month+tick_stage:elevation+tick_stage:month+tick_stage:location+elevation:location	tick_stage, elevation	0.31
betad~tick_stage+ location+elevation+tick_stage:elevation:month+tick_stage:elevation+tick_stage:month+tick_stage:location+elevation:location	tick_stage, location	0.28
betad~tick_stage+ location+elevation+tick_stage:elevation+tick_stage:month+tick_stage:location+elevation:location	tick_stage, location	0.24
betad~tick_stage+location+elevation+tick_stage:elevation+ tick_stage:location+elevation:location	tick_stage, location	0.21
betad~tick_stage+location+ elevation+ tick_stage:elevation	tick_stage	0.17
betad~tick_stage+elevation+ elevation:tick_stage	tick_stage	0.13
betad~tick_stage+elevation	tick_stage	0.11
**betad~tick_stage**	**tick_stage**	**0.08**

Table S3: Associations between the ecological variables and the group dispersion of tick microbiota diversity. As it can only be performed with one variable at a time, we corrected for multiple testing with Hochberg's correction [11].

Diversity index	Variable		Mean of squares	F-value	Df	Pr (>F)	Adjusted Pr (>F)
Bray-Curtis	**elevation**	**Groups**	**0.06**	**5.9**	**8**	**< 0.001**	**<0.001**
		**Residuals**	**0.01**		**70**		
	**location**	**Groups**	**0.06**	**5.3**	**2**	**0.007**	**0.02**
		**Residuals**	**0.01**		**76**		
	tick_stage	Groups	0.01	0.81	2	0.44	0.45
		Residuals	0.01		76		
	month	Groups	0.01	0.93	2	0.40	0.45
		Residuals	0.01		76		
Jaccard	**elevation**	**Groups**	**0.04**	**11.44**	**8**	**< 0.001**	**< 0.001**
		**Residuals**	**0.01**		**70**		
	location	Groups	0.01	3.48	2	0.04	0.11
		Residuals	0.01		76		
	tick_stage	Groups	0.01	2.54	2	0.09	0.17
		Residuals	0.01		76		
	month	Groups	0.01	1.67	2	0.19	0.19
		Residuals	0.01		76		
Weighted Unifrac	**elevation**	**Groups**	**0.02**	**3.11**	**8**	**0.01**	**0.02**
		Residuals	0.01		70		
	location	Groups	0.02	2.68	2	0.07	0.22
		Residuals	0.01		76		
	tick_stage	Groups	0.01	0.56	2	0.57	0.57
		Residuals	0.01		76		
	month	Groups	0.01	1.20	2	0.31	0.56
		Residuals	0.01		76		

S3: Supplementary methods and results of random forest analysis

We used Breiman's Random Forest Classifier approach with the package *randomForest* [12] to assess how well our variables of interested delimit different categories. In other words, this test assesses whether it can be predicted from the bacterial community composition whether it originates from a tick collected in a specific month, elevation or tick life stage/sex. We ran the algorithm for each of the previously mentioned variables and measured the predictive accuracy by out-of-bag error rate [12].

The random forest analysis provided an out-of-bag score of 43.0% for elevation (classification error for low elevation 0.11, for middle elevation 0.83 and for high elevation 1.00), 34.2% for sampling month (classification error for June 0.72, for July 0.17 and for August 1.00), 27.5% for tick stage/sex (classification error for females 0.19, for males 0.41 and for nymphs 0.80) and 32.0 % for sampling location (classification error for Sagogn 0.29, for Roedels 0.55 and for Passug 1.00), which corresponds to the expected percentages based on random guessing. It suggests that none of these variables can be reliably predicted based on OTU composition.

S4: Supplementary methods and results of tick population genetic and phylosymbiosis analyses

To calculate genetic distances among individual ticks, neutral genetic diversity was determined as described in Aivelo et al. [7]. In short, we genotyped ticks at 11 microsatellite markers in two multiplexed amplifications and used the package *poppr* [13] in R 3.4.1 [14] to calculate Prevosti's distance between individual ticks [15].

To test for phylosymbiosis, we analyzed whether the neutral genetic distance between ticks explains differences in their bacterial communities. The association between the genetic distances among individual ticks and genetic distances of their bacterial microbiotas was calculated in three different ways: First, we correlated the dissimilarities of tick microbiota (wUF) and genetic distances among ticks and fitted loess (local polynomial regression lines [16]) with $\alpha = 0.2$. Second, we tested for an association between the microbiota weighted UniFrac matrix and the tick genetic distance matrix using Mantel test [17]. Third, we compared trees created based on tick neutral genetic distances and tree based on tick microbiota UniFrac distances by calculating the Matching Split Distance [18, 19] for unrooted binary tree. Matching Split Distance compares tree branches implied by a pair of splits between the two trees and calculates the number of taxa that must be moved from one branch to

another to make two splits identical. UniFrac-based trees with branch tip labels randomly assigned 10,000 times were used as a null models for this analysis.

No evidence for phylosymbiosis between ticks and their bacterial microbiota community was found across tests. Pairwise neutral genetic distances between individual ticks did not correlate with the dissimilarities in within-tick microbiota composition (i.e., weighted UniFrac) (LOESS curves: pseudo-R^2 = 0.40), suggesting that neutral genetic differences among ticks do not explain variation in within-tick community composition. Likewise, the comparison of tick genetic distance and within-tick microbial community distance matrices did not show a significant association (Mantel test: r = -0.05, p = 0.80). Finally, the phylogenetic tree of sampled ticks and the tree based on the dissimilarities of their microbiota were not significantly similar (Matching Split Distance: required number of substitutions is 583, p = 0.32 compared to null model), meaning that differentiation in tick microbiota did not reflect the genetic differentiation in ticks.

Tick population differentiation across sites, elevations and tick stage/sex were evaluated by calculating pairwise F_{ST} values according to Nei [20] with the package *hierfstat* [21]. We performed Mantel tests [17] with 9999 permutations in the package *ade4* [22] to test for isolation by distance and isolation by elevation. There was no evidence for isolation by distance or isolation by elevation in tick populations (Mantel tests (IBD: r = - 0.29, $p_{simulated}$ = 0.95; IBE: r = 0.02, $p_{simulated}$ = 0.44).

Table S4a: Pairwise F_{ST} values based on tick stage/sex.

	F	M
M	- 0.001	
N	0.009	0.008

Table S4b: Pairwise F_{ST} values based on site

	Rodels	Tomils	Feldis	Sagogn	Flims	Ruschein	Passug	Castiel
Tomils	0.022							
Feldis	0.064	0.017						
Sagogn	0.022	-0.001	-0.044					
Flims	0.015	-0.001	-0.071	-0.017				
Ruschein	0.025	0.020	0.030	-0.008	0.001			
Passug	0.035	0.003	-0.054	0.001	0.002	-0.004		
Castiel	0.042	0.238	0.041	-0.001	0.009	0.036	0.036	
Praden	0.010	0.034	0.012	0.005	-0.025	-0.079	0.059	0.032

Table S4c: Pairwise F_{ST} values based on elevation

	High	Low
Low	0.0033	
Middle	0.0184	- 0.028

Based on the neutral microsatellite markers, there was thus no obvious differentiation in tick populations due to either geographic or altitudinal distances. This suggests that the difference in microbiota across life stage/sex is not due to different dispersal or differences in population composition of nymph, males or females or along elevational gradient. Furthermore, this lack of population structure could explain why there was no evidence of phylosymbiosis between tick populations and microbiotal structure.

S5: Supplementary results of influences of ecological processes on community turnover

Table S5: The median values and interquartile range of βNTI and Bray-Curtis-based Raup-Crick for the whole sample and divided by elevation classes and tick stage/sex.

	Whole sample	Elevation			Tick stage/sex		
		Low	Middle	High	Female	Male	Nymph
βNTI	-0.29<br>*-1.20 – 0.80*	– 0.32<br>*-1.16 – 0.69*	– 0.20<br>*– 1.09 – 1.10*	1.09<br>*0.40 – 1.30*	-0.71<br>*-1.55 – 0.35*	-0.20<br>*-1.09 – 1.10*	0.78<br>*-0.10 – 1.15*
Raup-Crick	0.51<br>*0.00 – 0.80*	0.49<br>*0.00 – 0.77*	0.47<br>*0.01 – 0.73*	0.28<br>*0.01 – 0.73*	0.19<br>*-0.34 – 0.62*	0.47<br>*0.01 – 0.73*	0.87<br>*0.42 – 0.97*

Table S6: The number of occurrences of each type of ecological processes in between-pair comparisons in all samples, between different sites, within sites in elevation and tick stage/sex categories. The numbers are crude numbers of between-pair comparisons. Rule describes how between pair comparisons are assigned to one of the five processes. Samples from high elevations and nymphs were excluded from between and within-sites comparisons due to low sample size per category, but they were included in 'All'.

Process		Rule	All	Between-site	Within-site				
				Total	Total	By elevation		By tick stage/sex	
Type	Name					Low	Middle	Female	Male
Deterministic									
	Variable selection	βNTI > 2	59	38	11	8	3	2	4
	Homogenous selection	βNTI < -2	273	157	36	23	13	13	2
Stochastic									
	Homogenizing dispersal	-2 < βNTI < 2 & BC-RC < -0.95	44	27	9	7	1	4	2
	Dispersal limitation	-2 < βNTI < 2 & BC-RC > 0.95	280	169	37	23	14	3	2
	Undominated	-2 < βNTI < 2 & -0.95 < BC-RC < 0.95	2 425	1405	390	285	105	178	63

S6: Sensitivity analysis

The sample distribution across elevations and tick life stages/sexes is uneven, as there were less nymph samples and samples from the highest elevations (Table 1). To demonstrate the robustness of our findings, we explored the sensitivity of our results to sample size variation. We re-analysed the final models presented in the main text without nymphs and samples from the highest elevations to assess whether these lower sample groups biased the results.

Overall, our results were robust to unequal sample sizes: no changes in significance were found in alpha diversity metrics (Table S7a) or in permutational ANOVA of beta diversity indices (Table S7b). For some models, the conditional R^2 slightly increased (e.g., Faith's diversity) and for some it decreased (e.g., Inverse Simpson). In contrast, the group dispersion analysis was more sensitive (Table S7c); specifically, location was no longer a significant factor in the Bray-Curtis model, whereas it became significant in the Jaccard model. For the weighted Uni-Frac model, elevation was no longer statistically significant.

The sensitivity analysis of random forest models provided an out-of-bag score of 36.3% for elevation (classification error for low elevation 0.04 and for middle elevation 0.95), 47.0% for sampling month (error for June 0.70, for July 0.07 and for August 1.00), 28.8% for tick life stage/sex (error for females 0.20 and for males 0.39) and 51.5 % for sampling location (error for Sagogn 0.57, for Rodels 0.21 and for Passug 1.00), which corresponds to the expected percentages based on random guessing, suggesting no effect of including or not including nymphs and samples from the high elevations on the results.

Sensitivity analysis also confirmed the robustness of the phylosymbiosis analysis. No evidence for phylosymbiosis between ticks and their bacterial microbiota community was found across tests. Pairwise neutral genetic distances between individual ticks did not correlate with the dissimilarities in within-tick microbiota composition (i.e., weighted UniFrac) (LOESS curves: pseudo-R^2 = 0.49). Likewise, the comparison of tick genetic distance and within-tick microbial community distance matrices did not show a significant association (Mantel test: $r = -0.01$, $p = 0.62$). Finally, the phylogenetic tree of sampled ticks and the tree based on the dissimilarities of their microbiota were not significantly similar (Matching Split Distance: required number of substitutions is 439, $p = 0.39$ compared to null model).

Overall, these sensitivity analyses demonstrate that the findings presented in the main text are robust and not biased by unequal sample sizes across elevations and tick life stages.

Table S7a: The final linear mixed models for alpha diversity measures without nymphs or samples from the highest elevations.

Measure	Variables	Value	Standard error	DF	t	p	cond. R^2
Inverse Simpson	Female intercept	7.58	1.31	62	5.78	< 0.001	0.09
	Male	2.65	1.53	62	2.09	0.04	
Faith's phylogenetic diversity	June 0 meter intercept	1.95	2.99	61	0.65	0.51	0.14
	Elevation	-0.003	0.0001	61	-2.16	0.03	
	Month	0.93	0.41	61	2.25	0.03	
NRI	Female intercept	-1.90	0.21	62	-9.3	< 0.001	0.11
	Male tick	0.86	0.30	62	2.87	0.01	
NTI	Female intercept	-2.10	0.19	62	-11.1	< 0.001	0.06
	Male tick	0.56	0.28	62	2.05	0.05	

Table S7b: Best models describing bacterial microbiota beta diversity without nymphs or samples from the highest elevations.

		Mean squares	Modelled F	DF	R^2	p
Bray-Curtis	tick stage/sex	0.71	3.03	1	0.05	<0.01
	residuals	0.23		64	0.96	
Jaccard	elevation	0.32	1.30	1	0.02	0.05
	tick stage/sex	0.46	1.90	1	0.03	0.01
	residuals	0.24		63	0.95	
weighted UniFrac	tick stage/sex	0.17	2.13	1	0.03	0.04
	residuals	0.08		64	0.97	

Table S7c: Associations between the ecological variables and the group dispersion of tick microbiota diversity without nymphs or samples from the highest elevations..

Diversity index	Variable		Mean of squares	F-value	Df	Pr (>F)	Adjusted Pr (>F)
Bray-Curtis	**elevation**	**Groups**	**0.04**	**3.43**	**5**	**< 0.001**	**0.03**
		**Residuals**	**0.01**		**60**		
	location	Groups	0.04	3.38	2	0.04	0.12
		Residuals	0.01		63		
	tick_stage	Groups	0.00	0.12	1	0.73	0.73
		Residuals	0.01		64		
	month	Groups	0.02	1.24	2	0.29	0.59
		Residuals	0.01		63		
Jaccard	**elevation**	**Groups**	**0.01**	**4.44**	**5**	**0.001**	**0.01**
		**Residuals**	**0.00**		**60**		
	**location**	**Groups**	**0.01**	**4.46**	**2**	**0.02**	**0.05**
		**Residuals**	**0.00**		**63**		
	tick_stage	Groups	0.01	1.95	1	0.17	0.17
		Residuals	0.00		64		
	month	Groups	0.01	2.75	2	0.07	0.15
		Residuals	0.00		63		
Weighted Unifrac	elevation	Groups	0.01	2.02	5	0.08	0.29
		Residuals	0.00		60		
	location	Groups	0.01	2.42	2	0.09	0.29
		Residuals	0.00		63		
	tick_stage	Groups	0.00	0.13	1	0.71	0.71
		Residuals	0.00		64		
	month	Groups	0.01	1.01	2	0.37	0.71
		Residuals	0.00		63		

Supplementary references

1. Jaccard P (1912) The distribution of the flora in the alpine zone. New Phytol 11:37–50. https://doi.org/10.1111/j.1469-8137.1912.tb05611.x

2. Ndiribe C, Pellissier L, Antonelli S, et al (2013) Phylogenetic plant community structure along elevation is lineage specific. Ecol Evol 3:4925–4939. https://doi.org/10.1002/ece3.868

3. Pellissier L, Alvarez N, Espíndola A, et al (2013) Phylogenetic alpha and beta diversities of butterfly communities correlate with climate in the western Swiss Alps. Ecography (Cop) 36:541–550. https://doi.org/10.1111/j.1600-0587.2012.07716.x

4. Pitteloud C, Descombes P, Sànchez-Moreno S, et al (2020) Contrasting responses of above- and below-ground herbivore communities along elevation. Oecologia 194:515–528. https://doi.org/10.1007/s00442-020-04778-7

5. Pellissier L, Niculita-Hirzel H, Dubuis A, et al (2014) Soil fungal communities of grasslands are environmentally structured at a regional scale in the Alps. Mol Ecol 23:4274–4290. https://doi.org/10.1111/mec.12854

6. Aivelo T, Norberg A, Tschirren B (2019) Bacterial microbiota composition of *Ixodes ricinus* ticks: the role of environmental variation, tick characteristics and microbial interactions. PeerJ 7:e8217. https://doi.org/10.1101/559245

7. García-Navas V, Sattler T, Schmid H, Ozgul A (2020) Temporal homogenization of functional and beta diversity in bird communities of the Swiss Alps. Divers Distrib 26:900–911. https://doi.org/10.1111/ddi.13076

8. Caporaso JG, Lauber CL, Walters WA, et al (2012) Ultra-high-throughput microbial community analysis on the Illumina HiSeq and MiSeq platforms. ISME J 6:1621–1624. https://doi.org/10.1038/ismej.2012.8

9. Apprill A, Mcnally S, Parsons R, Weber L (2015) Minor revision to V4 region SSU rRNA 806R gene primer greatly increases detection of SAR11 bacterioplankton. Aquat Microb Ecol 75:129–137. https://doi.org/10.3354/ame01753

10. Walters W, Hyde ER, Berg-lyons D, et al (2015) Improved bacterial 16S rRNA gene (V4 and V4-5) and fungal internal transcribed spacer marker gene primers for microbial community surveys. mSystems 1:e0009-15. https://doi.org/10.1128/mSystems.00009-15.Editor

11. Hochberg Y (1988) A sharper Bonferroni test for multiple tests of significance. Biometrika 75:800–802. https://doi.org/10.2307/2336325

12. Breiman L (2001) Random Forests. Mach Learn 45:5–32

13. Kamvar ZN, Tabima JF, Grünwald NJ (2014) Poppr: an R package for genetic analysis of populations with clonal, partially clonal, and/or sexual reproduction. PeerJ 2:e281. https://doi.org/10.7717/peerj.281

14. Team RC (2013) R: A language and environment for statistical computing

15. Prevosti A, Ocaña J, Alonso G (1975) Distances between populations of Drosophila subobscura, based on chromosome arrangement frequencies. Theor Appl Genet 45:231–241. https://doi.org/10.1007/BF00831894

16. Cleveland WS, Grosse E, Shyu WM (1992) Local Regression Models. In: Chambers JM, Hastie TJ (eds) Statistical Models in S, 1st ed. Routledge, Boca Raton, LA, USA, p 624

17. Mantel N (1967) The detection of disease clustering and a generalized regression approach. Cancer Res 27:209–220. https://doi.org/10.5694/j.1326-5377.1952.tb83832.x

18. Bogdanowicz D, Giaro K, Wróbel B (2012) TreeCmp: Comparison of trees in polynomial time. Evol Bioinforma 2012:475–487. https://doi.org/10.4137/EBO.S9657

19. Lin Y, Rajan V, Moret BME (2012) A metric for phylogenetic trees based on matching. IEEE/ACM Trans Comput Biol Bioinforma 9:1014–1022. https://doi.org/10.1109/TCBB.2011.157

20. Nei M (1987) Molecular Evolutionary Genetics. Columbia University Press, New York, USA

21. Goudet J, Jombart T, Kamvar ZN, et al (2020) hierfstat

22. Dray S, Dufour AB (2007) The ade4 package: Implementing the duality diagram for ecologists. J Stat Softw 22:1–20. https://doi.org/10.18637/jss.v022.i04
